# Facilitation of Enrollment onto Cancer Clinical Trials Using a Novel Navigator-Assisted Program: A Cross-Sectional Study

**DOI:** 10.3390/curroncol31110526

**Published:** 2024-11-14

**Authors:** Mahmoud Hossami, Rhonda Abdel-Nabi, Farwa Zaib, Kayla Touma, Renee Nassar, Sanghyuk Claire Rim, Milica Paunic, Olla Hilal, Pratham Gupta, Roaa Hirmiz, Michael Touma, Govana Sadik, Emmanuel Akingbade, Depen Sharma, Swati Kalia, Rija Fatima, Anthony Luginaah, Ibrahim Mohamed, Rong Luo, Megan Delisle, Caroline Hamm

**Affiliations:** 1 Faculty of Biomedical Science, University of Windsor, Windsor, ON N9B 3P4, Canada; hossamim@uwindsor.ca (M.H.); abdelnr@uwindsor.ca (R.A.-N.); toumak@uwindsor.ca (K.T.); sadikg@uwindsor.ca (G.S.);; 2 Department of Oncology, Western University, Windsor Campus, Windsor, ON N8W 2X3, Canada; fzaib2025@meds.uwo.ca (F.Z.); rims@uwindsor.ca (S.C.R.);; 3 Clinical Trials Navigator Inc., Windsor, ON N8W 2X3, Canada; 4 School of Medicine, Griffith University, Gold Coast, QLD 4111, Australia; 5 Department of Surgery, University of Manitoba, Winnipeg, MB R3E 0V9, Canada; 6 Paul Albrechtsen CancerCare Manitoba Research Institute, Winnipeg, MB R3E 0V9, Canada

**Keywords:** clinical trials accrual, navigator, cancer

## Abstract

Introduction: Clinical trials are essential to the advancement of clinical therapies that improve the outcomes of people with cancer. However, enrollment in clinical trials remains a challenge. The Clinical Trial Navigator [CTN] Program was designed to address the current gap in the cancer care journey by assisting with the clinical trials search process. Methods: Between March 2019 and July 2024, applicants of the CTN program included people with cancer, their family members, and/or their care team. Applicants entered the CTN program through a REDCap^®^ survey that collected the patient’s medical history. A final curated list of potential clinical trials was provided to the applicant. Metrics of success included clinical trial referral and enrollment, and we examined the factors that impacted these outcomes. Results: A total of 445 people with cancer applied to the CTN program during the study. Of the 262 patients with referral and enrollment information, a trial referral occurred in 27.5% [n = 72]. Of the 72 patients who were referred to a clinical trial, 13 [18.1%] were enrolled, 9 [12.5%] are pending enrollment, and 50 [69.4%] were not enrolled. We identified a potential trial for 88% of applicants, with a median of one potential trial per patient. Physicians were highly involved as applicants. Interpretation: The CTN program is successful in searching for clinical trials for people with cancer. Ongoing implementation into other Canadian sites, assessments of patient-reported outcomes, website and social media campaigns, and research into the factors that impact referral and enrollment are underway.

## 1. Introduction

Clinical trials are essential to the advancement of clinical therapies and improve the outcomes of people with cancer. However, the rate of trial participation in cancer clinical trials is approximately 5% in Canada [1,2]. This continues to be a focus of concern for most clinical trial consortiums, research groups, and pharmaceutical companies [1,3,4,5,6,7,8,9,10,11].

Frameworks to address challenges with clinical trials accrual have been developed and categorized into structural [clinician and patient], attitudinal [physician and patient], and demographic/socioeconomic challenges [4,12,13]. Lack of physician time and structural support to identify appropriate trials for patients has been reported in the literature and continues to be a primary barrier [9,14,15,16,17,18]. Geographic barriers exist in Canada for clinical trial participation, with smaller and more remote locations reporting a clinical trial enrollment rate of only 3–5% [2,5,9,19,20]. Smaller cancer centres report an inability to conduct as many clinical trials as large academic centres for reasons such as site selection, the availability of necessary technology, and support for trial complexity [21]. Up to 85% of people living in Canada are impacted by this disparity of access to clinical trials because they live outside of large urban areas [22,23]. Patients are often referred to clinical trials search engines by their healthcare team; however, challenges with clinical trials search engines have also been previously reported [12,13,24,25,26].

The Clinical Trials Navigator [CTN] Program was established in Canada in 2019 as a novel solution to address some of these major barriers to clinical trials accrual [27]. The CTN program was established in 2019 in collaboration with the Canadian Cancer Clinical Trials Network (3CTN).

We previously published the short-term results of the CTN program between 2019 and 2020 [28]. During this first year, 118 people with cancer applied to this service. This article reports the updated results of the CTN program. We hypothesized that this navigator-assisted CTN program improves clinical trial referral and enrollment.

## 2. Materials and Methods

This study was an observational study reported using the STROBE guidelines [29]. This research obtained approval by the Research Ethics Board at Windsor Reginal Hospital #22-439, Category A approval.

The CTN program was designed and implemented at a pilot site: a community hospital located in Windsor, Ontario, Canada, in 2019. The Windsor Cancer Program is located 200 km from the closest cancer program and 400 km from the leading cancer program in the province.

Applicants across Canada were made aware of the CTN program through the Canadian Cancer Clinical Trials Network [3CTN] website [2], presentations at 3CTN Annual Stakeholder meetings, word of mouth, and CTN posters displayed at the host site [Windsor Regional Hospital]. The CTN was also accessible through the CTN emails: clinicaltrialsnavigator@wrh.on.ca and info@clinicaltrialsnav.com.

To access the CTN program, a REDCap^®^ survey was completed by an applicant [a person with cancer, their caregiver, or a member of the healthcare team] or a CTN program staff member on the request of a client. REDCAp^®^ v13.4.12 is a software licensed to the University of Windsor. An Equity, Diversity, and Inclusion [EDI] survey was added on 2 December 2022. Once completed, it was followed by one of 107 cancer-specific surveys, based on American Society of Clinical Oncology Cancer.Net fact sheets [30]. This survey captured detailed information on a person’s cancer medical history. This allowed the team to provide a reliable, curated list of potential trials for patients. The information was then de-identified and provided to the navigators, who performed clinical trial searches using the novel Master Lists developed by the CTN program.

The clinical trials navigators required a university degree or were placement students in master’s or clinical trials management courses. They were required to have a proficiency in reading scientific literature, as this is required to interpret the clinicaltrials.gov website [26,31]. They underwent a comprehensive onboarding program led by team staff, and weekly quality improvement sessions regarding their clinical trials searches were undertaken. Each navigator led a maximum of three disease sites to allow the development of disease-site expertise.

The cancer-specific Master Lists were developed and maintained by the navigators who implemented a search across five clinical trials search engines [ClinicalTrials.gov [32], Canadian Cancer Trials, Clinical Trials Ontario, 3CTN, and OncoQuebec]. Canadian Cancer Clinical Trials was sunsetted by the Canadian Cancer Society in July 2023. These Master Lists were developed to overcome some of the challenges of clinical trials search engines that were identified during the early phases of the CTN program. These challenges were described in a previous paper [33].

Potential trials were drawn from the Master Lists, reviewed by a CTN oncologist, and sent to the applicant for review with their treating oncologist. If deemed appropriate by the treating oncologist, the patient was subsequently referred to the clinical trial.

### 2.1. Data Sources and Measurements

Applicants signed an e-consent form through REDCap^®^ [Research Electronic Data Capture]. REDCap^®^ is a secure, web-based software platform used to support data capture for research studies. REDCap^®^ survey tools were used, and data were stored on the University of Windsor REDCap^®^ server.

Between 3 March 2019 and 25 July 2024, follow-up metrics were collected. These included referrals to and enrollment onto clinical trials. A follow-up survey was sent one month after the delivery of the list of potential trials. Applicants could offer if they had been referred to and/or enrolled into a clinical trial. As well, at the pilot site, we had access to patient medical charts. These charts were reviewed to collect referral and enrollment data. This information was entered into the REDCap^®^ Follow-up Survey database by the lead navigator. Analysis of this database was performed by the lead navigator, the principal investigator, and the statistician.

Data were available from 6 February 2022 to 25 July 2024 regarding particular applicants, with 257 people with cancer participating in the CTN program. Applicants identified as self, family, physician, or other. Information on the applicant was not available before 6 February 2022.

Type of cancer, stage, Karnovsky performance status, referral to clinical trial, and number of previous lines of treatment were analyzed. Follow-up data on referrals to and enrollment onto clinical trials were mostly restricted to patients from the pilot site, as this was the only site that had an on-site navigator with access to patient medical records. Applicants not from the pilot site had the option of volunteering follow-up data; however, limited data were offered.

Time of application to the CTN program to death was recorded only at the host site, as we were unable to collect these data reliably from remote applicants.

### 2.2. Statistical Methods

We compared the cancer diagnosis of the people registered into the CTN program to Canadian cancer statistics using a one-sample proportion test. To explore potential differences in the likelihood of successful referral and enrollment in clinical trials, we utilized Fisher’s exact test. Patient survival was captured through chart review at the host site only, up to 25 July 2024.

## 3. Results

### Demographic Characteristics

Between 3 March 2019 and 25 July 2024, 445 people with cancer in Canada applied to the CTN program [Table 1].

Previous lines of therapy per patient were available for 218 patients. Notably, 58 patients [26.6%] had no prior treatment, 74 [33.9%] had one prior line of treatment, 45 [20.6%] had 2 lines of therapy, 36 [16.5%] had 3–5 lines, and 5 [2.3%] had 6 or more lines of treatment.

Type of applicant was available for 257 people from 6 February 2022 to 25 July 2024. Of the applications to the CTN program, 106 [41.2%] were physicians, 89 [34.6%] were self-applicants, and 30 [11.7%] were family members. Thirty-two [12.5%] applicants were from other sources.

Since the EDI survey was launched on 2 December 2022, 185 applicants have completed it. Of these, 5 [2.7%] self-identified as Indigenous; 3 [1.6%] as gay/lesbian; 12 [6.5%] preferred not to answer; 140 [75.7%] identified as heterosexual; and 25 [13.5%] submitted other answers. We have ethnicity data on 234 applicants, and we identified the following: 183 were Caucasian [78.2%], 1 was American Indian [0.4%], 8 were Middle Eastern [3.4%], 18 were Asian [7.7%], 4 were Black/African American [1.7%], 3 were Latino/Spanish [1.3%], 15 came under Other (which included Indigenous) [6.4%], and 6 preferred not to answer [2.6%]

We collected information on types of potential trials available and this information is available for 425 patients. A median of one potential clinical trial was identified for applicants, with a range of 0–25 potential trials per applicant. Zero trials were identified for 51 [12.0%] applicants, and at least one potential trial was available for 374 [88%] applicants. A total of 1422 potential trials were identified for 425 applicants [Table 2].

The most common cancer types presented by the applicants were breast, brain, and colorectal [Table 1]. Lymphoma, brain, and pancreatic cancer were more highly represented in our data set compared to Canadian statistics. Prostate and lung cancers were under-represented [Table 3].

**Figure 1:**
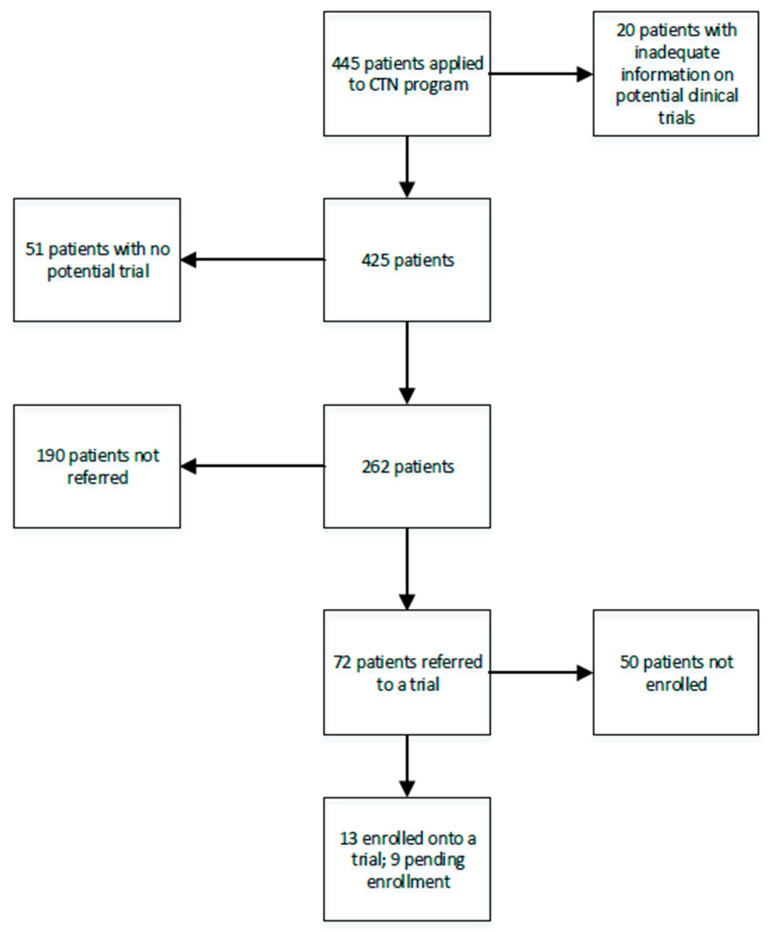
Flow diagram of patients who applied to the CTN program.

We have information on 262 patients regarding referrals and enrollment to clinical trials identified through the CTN program. Of these, 72 of 262 [27.5%] were referred to a CTN-suggested potential clinical trial by their treating oncologist. Of those referred, 13 of 72 [18.1%] were enrolled onto a clinical trial, 9 [12.5%] are pending enrollment, and 50 [69.4%] were not enrolled [Figure 1].

We examined the differences in characteristics between people with cancer who were or were not referred by their oncologist [Table 4] to a clinical trial. Our analysis did not reveal any statistically significant differences in referral patterns by cancer type, stage, or lines of prior therapy.

Data on survival are only available from the host site at this time. Survival from the time of application to the CTN program to last follow-up on 25 July 2024 was recorded. The median time from application to the CTN program to death was recorded to be 5.9 months [range: 0.3–46.5 months].

**Figure 2:**
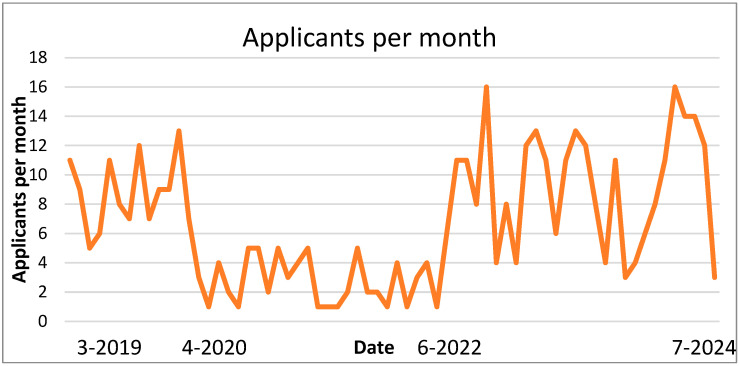
Applicants to the CTN program per month.

The effect of the pandemic is demonstrated in Figure 2. A dramatic drop in applicants to the program is demonstrated from the onset of the COVID-19 pandemic in the spring of 2020. Recovery was delayed and required a restructuring effort to realize recovery.

## 4. Discussion

The CTN program was successful in facilitating the referral of 27.5% of people with cancer for whom a potential clinical trial was identified, and 18.1% of those referred enrolled into a clinical trial. When compared to published clinical trial enrollment rates averaging around 5%, this suggests that the CTN program is a successful tool for improving clinical trial referral and enrollment. Those patients who enrolled through the CTN would have otherwise not enrolled through currently available pathways.

The CTN program is a ‘patient-driven approach’ to clinical trials [12]. Most people with cancer treated in centres outside large academic hospitals require a support structure to navigate referral and enrollment onto a clinical trial [22,23].

Structural barriers to patients include challenging clinical trials search engines [12,13,24,25,31]. The most commonly used search engine is written so that an experienced reader of scientific literature would be able to interpret it [34]. This would disadvantage most patients. It has been demonstrated that patients, especially those with poor cancer literacy, are challenged when it comes to using clinical trials search engines [13,26]. In one study, the average person identified less than 20% of the appropriate clinical trials [13]. The CTN program addresses this gap in the heathcare experience.

The CTN program also addresses physician barriers to clinical trial referrals. Physician barriers such as lack of time and lack of support to identify appropriate clinical trials have been identified as long-standing challenges [4,9,15,16,17]. Physician involvement in 41.2% of the applications demonstrates that the CTN program is filling a gap in the healthcare continuum.

This study helps to resolve a well-recognized challenge in clinical trials enrollment: the lack of available clinical trials for patients who are interested in clinical trials [4,35,36,37,38,39]. In our study, using Master Lists to search for eligible clinical trials, we could identify a potential clinical trial for 88% of interested patients. This demonstrates that that the CTN program fills this gap in the current clinical trials system.

Patients were provided with a median of one potential trial. This supports the highly curated clinical trials search provided by the CTN program. We are able to provide such a curated list of potential trials to the applicants because of the detailed intake form that is used and the structure of the CTN program, which uses oncologist review. This provides a complete and yet not overwhelming list of potential trials to the applicant. The success of this process is demonstrated in the fact that 18.1% of those referred were successfully enrolled onto the proposed clinical trial.

In the literature, a lack of available clinical trials is commonly reported, with 33–60% of people with cancer being unable to enroll in a clinical trial due to a lack of trial availability [4,21,35,36,40]. This was not reflected in the CTN program, where we identified a potential clinical trial for 88% of applicants. Most of the current literature speaks to the lack of clinical trials at the patient’s cancer treatment site. The CTN program searches outside of the patient treatment site and is able to identify potential clinical trials for most patients, underlining the need for this type of service for patients. Challenges remain with subsequent referrals and enrollments.

In terms of cancer type, we compared our findings with Canadian cancer statistics, revealing notable disparities in the representation of certain cancer types among people referred to the CTN program. Specifically, we observed that rare cancers such as brain, lymphoma, and pancreatic were significantly overrepresented in the application to the CTN program [13.8%, 5.76%, and 5.3%, respectively] compared to the Canadian population [0.9%, 1.0%, and 0.5%, respectively], while common cancers such as prostate cancer were present in 4.6% of our study cohort as compared to 19.2% of the Canadian cancer population. This pattern potentially exists due to the high motivation of people with rare cancers to enroll in clinical trials, as effective therapies are not as available. Although groups with rare cancers are considered small, they make up a significant population of people searching for clinical trials. The overall participation by cancer type was similar to a previous study on the clinical trials matching program by the American Cancer Society, as well as a recent report from Safran et al., which identified triple-negative breast cancer patients as using clinical trials searches more frequently [28,41,42].

Challenges in our study include the lack of a complete data set. Applicants did not always complete all questions in the intake and follow-up forms, which led to a smaller data set. To address these challenges, during our expansion, we will be placing a navigator at each of our expansion sites to collect full demographic and follow-up information. We are currently expanding to more cancer centres in Canada.

The COVID-19 pandemic also affected the CTN program, as most oncology clinical trials were significantly impacted globally between 2020 and 2021 [43]. Recovery is ongoing in many clinical trial sites.

Various programs have been developed to address the barriers to clinical trials accrual. Decentralized clinical trials and navigator programs are some of these initiatives [3,44]. The use of a navigator has previously been demonstrated to be successful in supporting patients in clinical trials accrual, including underserved populations [41,45]. Programs such as the CTN program are an important addition to the infrastructure needed to address the challenges of clinical trials accrual.

Expansion and implementation into other cancer centres is ongoing, with the installation of a CTN navigator at each site. This will raise awareness of the program and improve our ability to collect accurate information on patients, as well as collect follow-up information. These assistants will also support people with cancer and the healthcare team in registering patients into the CTN program. We are now collecting patient-reported outcomes in order to evaluate and improve the CTN program. We are examining the factors that led to successful referral and enrollment. Our website should be available shortly and will have a social media campaign to accompany this. We are working with partners to develop a smartphone app to be used by CTN navigators and applicants that will improve patient experience with the program, and we are investigating the use of Artificial Intelligence in this program.

## 5. Conclusions

Improving clinical trials accrual is critical to the advancement of clinical therapeutics. The CTN program addresses both physician and patient structural barriers. The lack of support in identifying potential clinical trials is a significant challenge in the landscape of cancer care, and is being addressed by the CTN program. The success of the CTN program is demonstrated by facilitating the referral of 27.5% of people with cancer to a potential clinical trial, with 18.1% of patients who were referred successfully enrolling in a trial. Ongoing implementation into other Canadian sites; websites, smartphone apps, and social media campaigns; as well as research into the factors that impact referral and enrollment are underway.

## Figures and Tables

**Table 1:** Demographic characteristics.

Demographic Characteristics		n = 445 [100%]
Age [years]	Median	59.5
	Range	16.6–90.6
Sex	Female	214 [48.1%]
	Male	173 [38.9%]
	Not reported	58 [13.0%]
Type of cancer	Breast	74 [16.6%]
	Brain	61 [13.8%]
	Colorectal	47 [10.6%]
	Lung	42 [9.4%]
	Other heme cancer	40 [9.0%]
	Lymphoma	26 [5.8%]
	Pancreatic	24 [5.3%]
	Prostate	21 [4.6%]
	Melanoma	17 [3.9%]
	Sarcoma	10 [2.3%]
	Other§	83 [18.7%]
Stage of Cancer	I/newly diagnosed	38 [8.5%]
	II	20 [4.5%]
	III	16 [3.6%]
	IV	345 [77.5%]
	Unknown	26 [5.8%]

Other§ included the following: adenoid cystic carcinoma, adrenal gland tumor, amyloidosis, anal cancer, appendix cancer, bile duct cancer [cholangiocarcinoma], bladder cancer, bone cancer [sarcoma of bone], Ewing sarcoma—childhood and adolescence, hereditary breast and ovarian cancer, kidney cancer, vulvar, and unknown. Other heme cancers included the following: leukemia—acute lymphocytic (ALL), leukemia—acute myeloid (AML), liver cancer, mesothelioma, multiple myeloma, myelodysplastic syndrome (MDS). Values May Not Add to 100% Due to Rounding.

**Table 2:** Results of potential trials identified.

	Median	Range
Phase I	0	0–15
Phase I/II	1	0–7
Phase II/III/IV	0	0–9
Total trials	1	0–25

**Table 3:** Prevalence of types of cancers referred to the CTN program vs. Canadian cancer statistics [n = 434 patients whose cancer type information is available].

	Canadian Statistics	CTN Program	*p* Value
Breast cancer	21.0%	16.6%	0.028
Prostate cancer	19.2%	4.6%	<0.001
Lung cancer	13.0%	9.4%	0.033
Colorectal cancer	12.0%	10.6%	0.410
Melanoma	6.0%	3.9%	0.084
Lymphoma	1.0%	5.76%	<0.001
Brain cancer	0.9%	13.8%	<0.001
Pancreatic cancer	0.5%	5.3%	<0.001
Other hematological cancer	NR	9.0%	
Sarcoma	NR	2.3%	
Other	NR	18.7%	

**Table 4:** Difference in cancer types among those who were referred to a clinical trial compared to those who were not.

	Yes n = 72 [%]	No n = 190 [%]	*p* Value
Cancer Type			0.406
Lung cancer	12 [16.7%]	16 [8.4%]	
Breast cancer	13 [18.1%]	39 [20.5%]	
Colorectal cancer	6 [8.3%]	10 [5.3%]	
Lymphoma	5 [6.9%]	16 [8.4%]	
Prostate cancer	7 [9.7%]	9 [4.7%]	
Other hematological cancers	5 [6.9%]	24 [12.6%]	
Pancreatic cancer	5 [6.9%]	9 [4.7%]	
Sarcoma	3 [4.2%]	6 [3.2%]	
Brain tumors	7 [9.7%]	23 [12.1%]	
Melanoma	2 [2.8%]	9 [4.7%]	
Other	7 [9.7%]	28 [14.7%]	
Missing diagnosis		1 [0.5% *]	

Cancer type did not affect referral patterns. * Values may not add to 100% due to rounding.

## Data Availability

All data used in this publication are available on REDCap through the University of Windsor. We will provide de-identified data to the publishers on request.
